# Imaging biomarkers of contrast-enhanced computed tomography predict survival in oesophageal cancer after definitive concurrent chemoradiotherapy

**DOI:** 10.1186/s13014-020-01699-w

**Published:** 2021-01-12

**Authors:** Chengbing Zeng, Tiantian Zhai, Jianzhou Chen, Longjia Guo, Baotian Huang, Hong Guo, Guozhi Liu, Tingting Zhuang, Weitong Liu, Ting Luo, Yanxuan Wu, Guobo Peng, Derui Li, Chuangzhen Chen

**Affiliations:** 1 Department of Radiation Oncology, Cancer Hospital of Shantou University Medical College, Shantou City, China; 2 Department of Oncology, CRUK/MRC Oxford Institute for Radiation Oncology, University of Oxford, Oxford, UK; 3 Department of Radiation Oncology, Zhongshan City People’s Hospital, Zhongshan City, China

**Keywords:** Oesophageal cancer, Imaging biomarker, Computed tomography, Prognosis

## Abstract

**Background:**

This study aimed to evaluate the predictive potential of contrast-enhanced computed tomography (CT)-based imaging biomarkers (IBMs) for the treatment outcomes of patients with oesophageal squamous cell carcinoma (OSCC) after definitive concurrent chemoradiotherapy (CCRT).

**Methods:**

Altogether, 154 patients with OSCC who underwent definitive CCRT were included in this retrospective study. All patients were randomised to the training cohort (n = 99) or the validation cohort (n = 55). Pre-treatment contrast-enhanced CT scans were obtained for all patients and used for the extraction of IBMs. An IBM score, was constructed by using the least absolute shrinkage and selection operator with Cox regression analysis, which was equal to the log-partial hazard of the Cox model in the training cohort and tested in the validation cohort. IBM nomograms were built based on IBM scores for individualised survival estimation. Finally, a decision curve analysis was performed to estimate the clinical usefulness of the nomograms.

**Results:**

Altogether, 96 IBMs were extracted from each contrast-enhanced CT scan. IBM scores were constructed from 11 CT-based IBMs for overall survival (OS) and 8 IBMs for progression-free survival (PFS), using the LASSO-Cox regression method in the training cohort. Multivariate analysis revealed that IBM score was an independent prognostic factor correlated with OS and PFS. In the training cohort, the C-indices of IBM scores were 0.734 (95% CI 0.664–0.804) and 0.658 (95% CI 0.587–0.729) for OS and PFS, respectively. In the validation cohort, C-indices were 0.672 (95% CI 0.578–0.766) and 0.666 (95% CI 0.574–0.758) for OS and PFS, respectively. Kaplan–Meier survival analysis showed a significant difference between risk subgroups in the training and validation cohorts. Decision curve analysis confirmed the clinical usefulness of the IBM score.

**Conclusions:**

The IBM score based on pre-treatment contrast-enhanced CT could predict the OS and PFS for patients with OSCC after definitive CCRT. Further multicentre studies with larger sample sizes are warranted.

## Background

Oesophageal cancer (OC) is one of the most common cancers globally and in 2018 its incidence and number of cancer-related deaths ranked seventh and sixth, respectively [1]. The management of OC typically involves multidisciplinary therapy, including definitive concurrent chemoradiotherapy (CCRT), which is the main standard treatment for oesophageal squamous cell carcinoma (OSCC) for medically unresectable tumours; and is also an option for resectable tumours. However, the outcomes of CCRT among these patients are still disappointing, with 3-year overall survival (OS) rates of 23–44.7% [2–5]. More than 50% of patients in the RTOG 85-01 trial and INT 0123 trial experienced locoregional disease progression [3, 5]. Patients with higher mortality risk following CCRT may benefit from more intensive primary treatment (e.g., planned radical surgery after CCRT), adjuvant therapy (e.g. chemotherapy), or more frequent follow-up. The application of these strategies requires the identification of patients with high mortality risk prospectively to achieve personalised management. Thus, to improve the overall survival of patients with OC after CCRT, it is crucial to predict the mortality risk of each individual patient.

Prediction of outcomes among patients with OC after CCRT remains an unmet clinical need. One of the most commonly used methodologies for prognostic evaluation in the clinic is the TNM staging system, which stratifies patients into different stages according to their tumour burden. Although the clinical stage system provides important insights for evaluating outcomes of patients with different stages, its role in survival prediction among patients with the same disease stage is non-significant. Indeed, previous studies have shown that the clinical stage system fails to predict heterogeneous outcomes of patients with locally advanced disease following CCRT [6–8]. A variety of other clinical factors and biomarkers have also been assessed for their prognostic potential [9–11]. Yet none of these factors have been widely used for the clinical stratification of patients and decision-making, as each of them has weaknesses and limitations.

By using current imaging techniques, quantitative imaging biomarkers (IBMs) could become an interesting way of assessing multiple cancer diagnosis and prognosis. These so-called radiomics could extract relevant information from commonly available images with a high throughput [12–14]. Previous studies have reported on the potential information to be gleaned from computed tomography (CT) IBMs in OC, and were able to assess and predict histopathological characteristics, treatment response, or survival outcome among patients with OC to some extent [15–19]. CT scans play an important role in the radiation treatment of OC, including diagnosis, staging, treatment planning, quality control, and follow-up. Non-contrast enhanced CT-based IBMs have been shown to be correlated with patient outcomes for a number of cancer types, including OSCC [18, 20]. However, the most commonly available imaging modalities for patients after undergoing definitive CCRT were not non-contrast enhanced CT scans but contrast-enhanced CT scans, which were performed during treatment planning. A previous study suggested that post-treatment IBMs extracted from contrast-enhanced CT images might have a correlation with OS in patients with OC who received definitive CCRT [16]. Although the sample size of this study was small and only included 26 cases of squamous cell carcinoma (SCC), it provided encouragement for the pursuit of further studies. The most common pathological type of OC in China is SCC, and radiotherapy is administered with a total dose of 60–66 Gy [21]. This range is much higher than the dose used in the standard treatment of OC via conventional fractionated radiotherapy (50.4 Gy). Oesophageal oedema is a common acute adverse event after definitive CCRT. There are fewer residual lesions that could be used for objective analysis or evaluation after definitive CCRT. Further, some patients with a complete response did not have residual lesions. It is unclear whether contrast-enhanced CT images obtained before treatment could serve as a feasible source for radiomics analysis in OSCC. Therefore, stronger evidence is needed in support of the implications for survival outcomes and the reliability of the methodology.

In this study, based on pre-treatment contrast-enhanced CT images, we sought to develop and validate an IBM score to predict OS and progression-free survival (PFS) for patients with OSCC and assess its value for individual OS and PFS estimation.

## Methods

### Patients

**Fig. 1:**
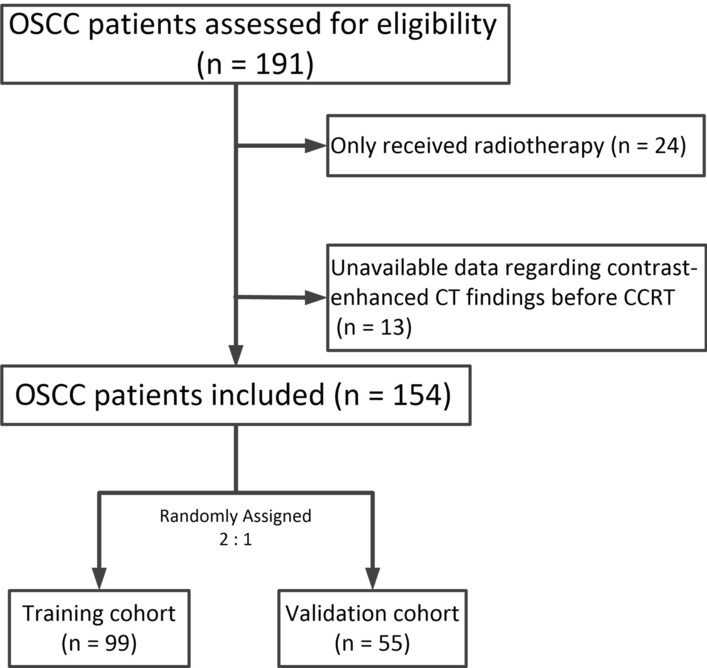
Flowchart of inclusion in the present study. OSCC, oesophageal squamous cell carcinoma; CCRT, concurrent chemoradiotherapy; CT, computed tomography

The protocol for this retrospective study was obtained from the local ethics and institutional review board. Approval and the need for informed consent had been waived. This study included patients with OC who underwent definitive CCRT at AAA between September 2009 and August 2015. The inclusion criteria were: (1) pathological diagnosis of OSCC; (2) primary tumour located in the cervical, upper thoracic, or middle thoracic oesophagus; and (3) contrast-enhanced CT scan findings, which were used in treatment planning before definitive CCRT. The exclusion criteria were: (1) patients who only received radiotherapy or chemotherapy; (2) prior surgery or administration of chest radiotherapy or chemotherapy. As shown in Fig. 1, the final study population consisted of 154 patients. All patients received intensity modulated radiation therapy (IMRT) combined with chemotherapy. Of these, 78 patients with OSCC were from a phase II prospective clinical study, using simultaneous modulated accelerated radiotherapy (SMART) combined with chemotherapy [22]. The 154 patients were randomly assigned into a training cohort (n = 99) and validation cohort (n = 55).

All patients underwent simulation CT scans for treatment planning. Seventy-eight patients underwent SMART, followed by radiation therapy with a prescribed dose of 66 Gy/30F, 5 days/week. Other patients underwent radiation therapy with a prescribed dose of 64/32F, 5 days/week. Most patients (90.9%) received concurrent chemotherapy based on the cisplatin and 5-fluorouracil (PF) regimen. The intensity of concurrent chemotherapy was relatively reduced among patients of advanced age or with poor performance status. Data regarding clinical characteristics of patients were collected in both cohorts, including age, sex, clinical stage, and tumour location. Dose-volume information for the primary tumour was collected from the radiotherapy planning system. Further details are shown in Table 1.

**Table 1:** Clinical characteristics of 154 patients with OSCC after definitive CCRT

Factors	Training cohort n (%)	Validation cohort n (%)	*p* value
Age, years			0.087^b^
Median (range)	61 (37–76)	58 (40–76)	
Sex			0.841^c^
Male	77 (77.8%)	42 (76.4%)	
Female	22 (22.2%)	13 (23.6%)	
Tumour location			0.325^c^
Cervical	16 (16.2%)	13 (23.6%)	
Upper	54 (54.5%)	31 (56.4%)	
Middle	29 (29.3%)	11 (20.0%)	
T stage^a^			0.469^c^
T2	11 (11.1%)	10 (18.2%)	
T3	46 (46.5%)	24 (43.6%)	
T4	42 (42.4%)	21 (38.2%)	
N stage^a^			0.655^c^
N0	36 (36.4%)	22 (40.0%)	
N1	63 (63.6%)	33 (60.0%)	
M stage^a^			0.668^c^
M0	87 (87.9%)	47 (85.5%)	
M1	12 (12.1%)	8 (14.5%)	
Clinical stage^a^			0.909^c^
II stage	29 (29.3%)	16 (29.1%)	
III stage	58 (58.6%)	31 (56.4%)	
IV stage	12 (12.1%)	8 (14.5%)	
Chemotherapy regimen			0.559^c^
RT with PF	91 (91.9%)	49 (89.1%)	
RT with other regimens	8 (8.1%)	6 (10.9%)	
Dose regimen			0.102^c^
2.2 Gy × 30 F	55 (55.6%)	23 (41.8%)	
2.0 Gy × 32 F	44 (44.4%)	32 (58.2%)	

OSCC, oesophageal squamous cell carcinoma; CCRT, concurrent chemoradiotherapy; AJCC, American Joint Committee on Cancer staging system (version 6.0th); RT, radiotherapy; PF, cisplatin and 5-fluorouracil

^a^American Joint Committee on Cancer (AJCC) staging system (version 6.0th)

^b^*p* value was analysed using the independent samples *t*-test

^c^*p* value was analysed using the chi-squared test

### Contrast-enhanced CT image acquisition

The CT scans of all patients were acquired (Philips Brilliance CT Big Bore Oncology Configuration, Cleveland, OH, USA; voxel size: 1.0 × 1.0 × 3.0 mm^3^ for 79 patients and 1.0 × 1.0 × 5.0 mm^3^ for 72 patients; convolution kernel: Philips Healthcare’s B), using a scanning voltage of 120 kVp with a slice thickness of 3–5 mm after an intravenous injection of 75 ml of 300 mg/mL iodinated contrast agent at a rate of 1.8–2 mL/sec with a pump injector (Medrad Stellant; Bayer, Beijing, China). The CT images were transmitted to the radiation therapy planning system (Eclipse Planning System version 10.0) via the DICOM 3.0 port.

### Region of interest (ROI) delineation and IBMs extracted

Pre-treatment contrast-enhanced CT scan images of patients were exported for analysis. The primary tumour was delineated by experienced radiation oncologists on the mediastinal window of the planning CT scan. IBMs were extracted by internal programming software using MATLAB R2016a (Mathworks, Natick, USA) and its toolbox. From the contrast-enhanced CT images of each patient, 96 IBMs were extracted, including the following types: (1) 24 CT intensity IBMs, describing the distribution of voxel parameter values in the volume of interest, such as the min, max and skewness of the primary tumour intensity; (2) 20 geometric IBMs that calculated the size and shape of the volume of interest, such as sphericity, volume, surface and long axis length; and (3) 52 texture IBMs, that described the difference in voxel density distribution of the three-dimensional contoured structure and consisted of four different matrices: grey level co-occurrence (GLCM) [23], grey level run-length (GLRLM) [24], neighbourhood grey-tone difference (NGTDM) [25], and grey level size-zone (GLSZM) matrices [26]. More details on the algorithms for IBM extraction and application have been discussed in previous studies [14, 27].

### Pre-selection Method and IBM score building

Because high correlations between most of the IBM variables were expected, in order to reduce the statistical probability of multi-collinearity, three rules were implemented to pre-select IBM variables for further analysis. First, IBM variables were assessed in the univariable analysis; variables with a *p* value less than 0.25 were used for the next analysis. Second, from highly correlated pairs of IBMs (i.e. the Pearson correlation coefficient r ≥ 0.8) variables with the higher *p* value in the Cox univariable analysis were omitted. Third, we performed the least absolute shrinkage and selection operator (LASSO) for the Cox regression model to select the most useful prognostic IBM variables from the potential predictors.[28]. The multiple-IBM-based scores (defined as the IBM scores), which were equal to the log-partial hazard of the Cox model, were calculated for each patient to reflect the risk of mortality or tumour progression and variance inflation factor (VIF) used to evaluate the collinearity among these final IBMs.

### IBM score performance and validation

As patients with OSCC were assigned into two cohorts, the performances of the IBM score were evaluated by the concordance indices (C-indices), respectively. The potential correlation of the IBM score with the OS and PFS for both the training and validation cohorts was assessed by using Kaplan–Meier survival curve analyses. Time-dependent receiver operating characteristic (ROC) curves were plotted for both the training and validation cohorts in term of OS and PFS. 95% confidence intervals were used as the confidence level on the ROC curves in this study. The optimal cut-off values of the ROC curves were determined using the Youden Indices (YIs) in the training cohort and patients were divided into high- and low IBM score subgroups; the thresholds of which were stratified by the maximum YIs. The same cut-off values were then applied to the validation cohort. Multivariable Cox proportional hazards analysis was used to assess the IBM score as an independent predictor by integrating clinical risk factors. In the training cohort, nomograms based on the IBM score were developed to assess individual patient-level probability estimates for the median survival time and 1-year, 3-year, and 5-year OS or PFS rates according to each patient’s unique combination of baseline characteristics. To estimate the clinical utility of the IBM nomograms, decision curve analysis (DCA) was used to quantify the net benefits at different threshold probabilities in both cohorts.

### Follow-up

The survival estimates mainly assessed in this study were OS and PFS. OS was defined as the time from the beginning of radiation therapy to death due to any cause or the last day of clinical follow-up, while PFS was defined as the time from the beginning of radiation therapy to first relapse at any site or death from any cause, whichever occurred first, or the last day of clinical follow-up.

### Statistical analysis

The clinical features of the patients in the two cohorts were compared using the independent t-test or chi-squared test, with a statistical significance level of 0.05 for 2-tailed test. All statistical analyses were performed using R version 3.6.0 (The R Foundation for Statistical Computing, Vienna, Austria) and SPSS version 23.0 (IBM Corp, Armonk, NY, USA). The LASSO algorithm was implemented using the glmnet package in the R environment [29]. The ROC and Kaplan–Meier curves were plotted using the *pROC* and *survminer* packages, respectively, in the R environment. Nomograms were constructed using the *rms* and *survival* packages in the R environment. The DCA curves were created using the *rmda* package in the R environment.

## Results

### Baseline clinical results

The clinical factors for the training and validation cohorts are listed in Table 1. Age was tested by independent t-test, and other clinical factors were tested by chi-squared test. No significant differences in patients’ clinical characteristics were found between the two cohorts. Of the 154 patients included in the study, 119 (77.3%) were men, and the median (interquartile range, IQR) age of all patients was 60 years (55–64.25 years). In the training cohort, the median (IQR) survival time for OS and PFS were 43 (19–59) and 36 (10–55) months, respectively. In the validation cohort, the median (IQR) survival time for OS and PFS were 37 (22–55) and 36 (12–54) months, respectively.

### IBM selection results

**Fig. 2:**
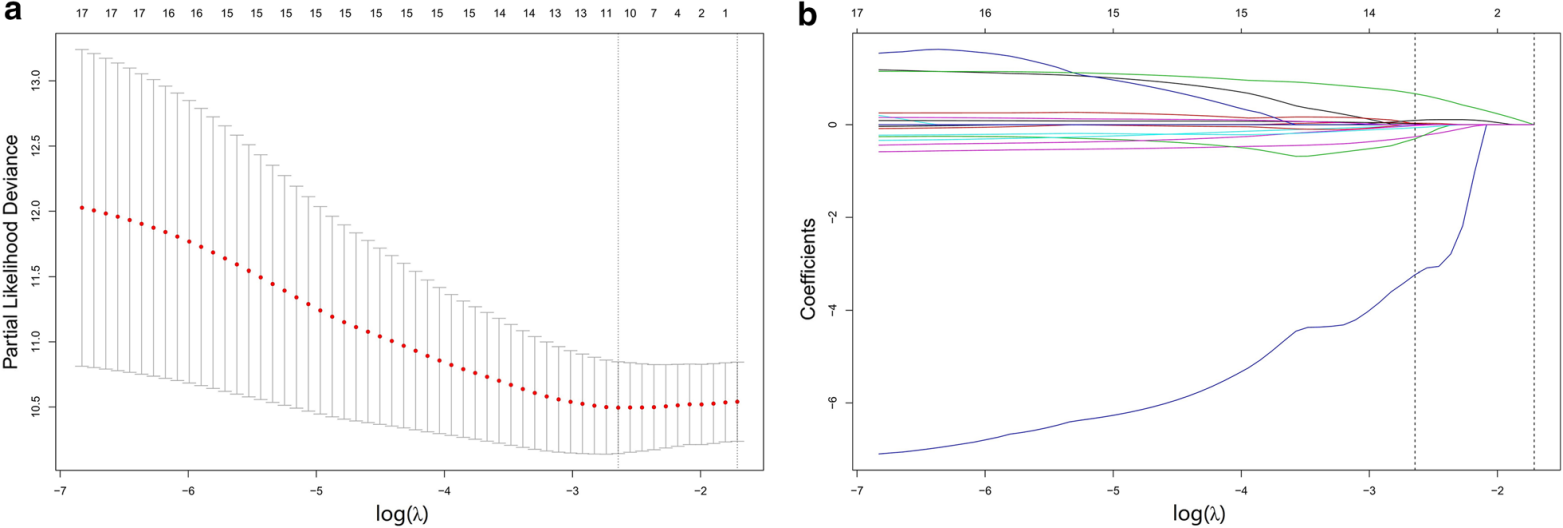
IBM selection using the LASSO-Cox regression model. **a** Ten-fold cross-validation was performed to select IBMs using LASSO method. At the optimal values of the tuning parameter (λ), the partial likelihood deviance curve was plotted versus log (λ). The dotted vertical line was set with minimum criteria, a λ value of 0.071127 with log (λ) of − 2.643291, where 11 IBMs were selected. **b** The coefficient profiles of 18 IBMs in the LASSO. The coefficient profile plot was generated against the log (λ) sequence. With ten-fold cross-validation, the dotted vertical line showed the non-zero coefficients selected, where eleven IBMs were selected. LASSO, least absolute shrinkage and selection operator; IBMs, imaging biomarkers

In the univariable analysis, 46 IBM variables were used and 18 IBMs remained after comparing the inter-variable correlations (Additional file 1: Table S1). 11 potential predictors with non-zero coefficients were selected in the LASSO Cox regression model. We plotted the partial likelihood deviance versus log (λ), where λ is the tuning parameter (Fig. 2). A dotted vertical line was drawn at log (λ) = –2.643, which corresponded to the best value λ = 0.071. The optimal tuning parameter resulted in 11 non-zero coefficients. With their corresponding coefficients in the LASSO-Cox model, the calculation formulas of IBM score for OS (Formula 1) was constructed as:

1 $$\begin{aligned} & {\text{IBM}}\,{\text{score}} = 0.019 \times {\text{Range}} - 3.231 \times {\text{Q}}975 - 0.260 \times {\text{Sphericity}} \\ & \quad + 0.093 \times {\text{Major\_axis}}\_{\text{length}} + 0.035 \times {\text{Maximum}}\_{\text{Probability}}\_{\text{GLCM}} \\ & \quad - 0.304 \, \times {\text{Sum}}\_{\text{of}}\_{\text{Square}}\_{\text{Variance}}\_{\text{GLCM}} - 0.014 \times {\text{Coarseness}}\_{\text{NGTDM}} \\ & \quad - 0.020 \times {\text{Contrast}}\_{\text{NGTDM}} + 0.020 \times {\text{Busyness}}\_{\text{NGTDM}} + 0.670 \\ & \quad \times {\text{Small}}\_{\text{Zone}}\_{\text{Emphasis}}\_{\text{GLSZM}}- 0.064 \times {\text{Zone}}\_{\text{percentage}}\_{\text{GLSZM}} + 2.5. \\ \end{aligned}$$

The constant value was 2.5, which was used to obtain IBM scores > 0 from the calculation formula. The VIFs of the 11 IBMs were acceptable, ranging from 1.150–3.403, indicating no collinearity problems (Additional file 1: Table S2).

The same analysis was used to select the IBMs which were associated with PFS in the training cohort. 32 IBM variables were preselected in the univariable analysis and 12 IBMs remained after comparing the inter-variable correlations (Additional file 1: Table S1). A dotted vertical line was drawn at log (λ) = − 2.702, which corresponded to the best value λ = 0.067 (Additional file 2: Figure S1a and Figure S1b). The optimal tuning parameter resulted in 8 non-zero coefficients. With their corresponding coefficients in the LASSO Cox model, the calculation formulas of IBM score for PFS (Formula 2) was constructed as:

2 $$\begin{aligned} & {\text{IBM}}\,{\text{score}} = - 0.663 \times {\text{Q}}75 - 0.234 \times {\text{Q}}975 - 0.246 \\ & \quad \times {\text{Volume}}\_{\text{Density}}- 0.01 \times {\text{Sphericity}} + 0.122 \\ & \quad \times {\text{Major}}\_{\text{axis}}\_{\text{length}} - 0.021 \times {\text{Contrast}}\_{\text{NGTDM}} \\ & \quad + 0.468\times {\text{Small}}\_{\text{Zone}}\_{\text{Emphasis}}\_{\text{GLSZM}} - 0.069\\& \quad \times {\text{Zone}}\_{\text{percentage}}\_{\text{GLSZM}} - 1.4. \end{aligned}$$

The VIFs of the 8 IBMs were acceptable, ranging from 1.266 to 4.524, indicating no collinearity problems (Additional file 1: Table S2).

### Performance of IBM score

**Fig. 3:**
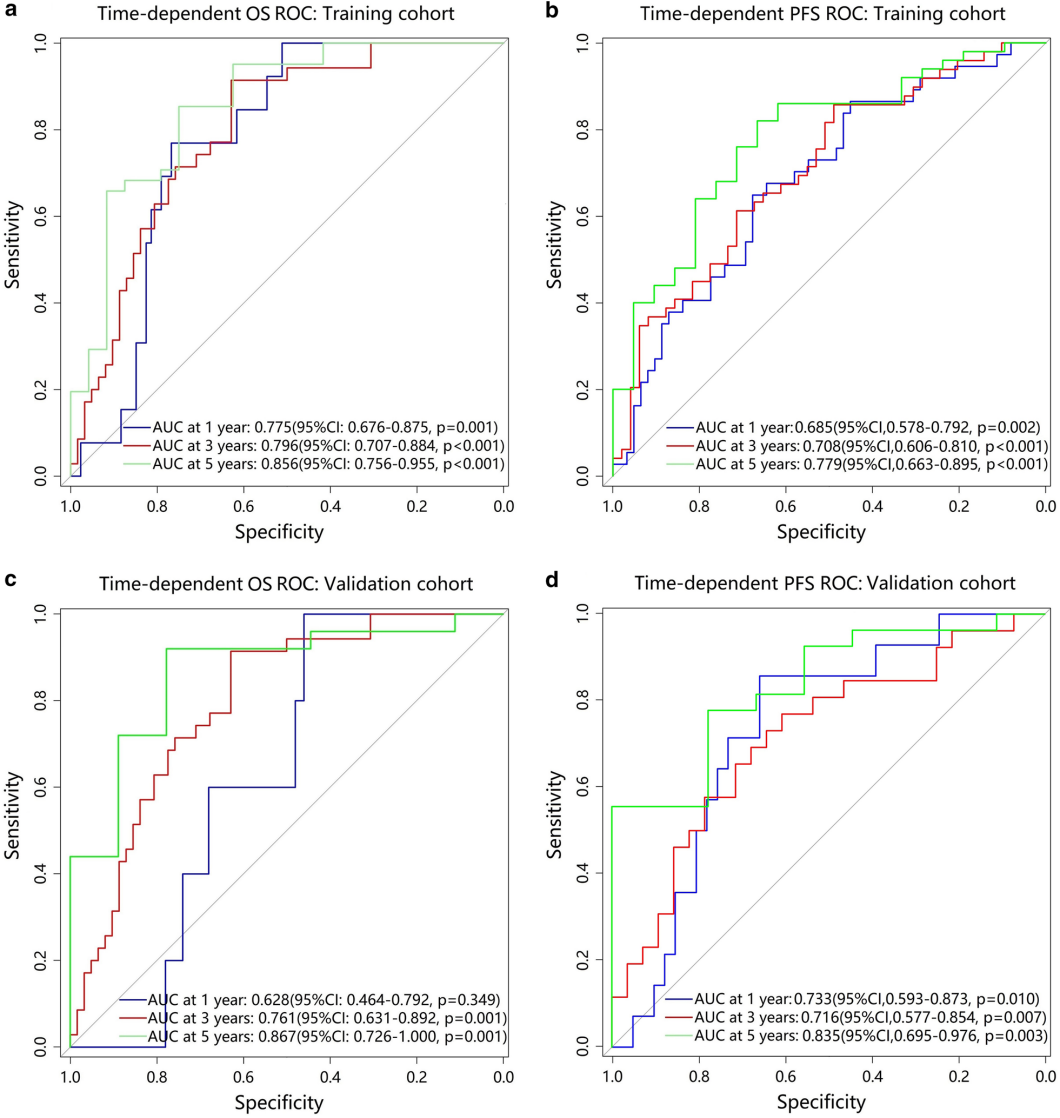
Time-dependent ROC curves for the performance of IBM scores in the training cohort and validation cohort. **a** Time-dependent OS ROC for training cohort; **b** Time-dependent PFS ROC for training cohort; **c** Time-dependent OS ROC for validation cohort; **d** Time-dependent PFS ROC for validation cohort. AUCs for 1, 3, 5 years were used for survival prediction. ROC, receiver operation characteristic; AUC, area under the curve; CI, confidence interval; OS, overall survival; PFS, progression-free survival; IBM, image biomarker

**Fig. 4:**
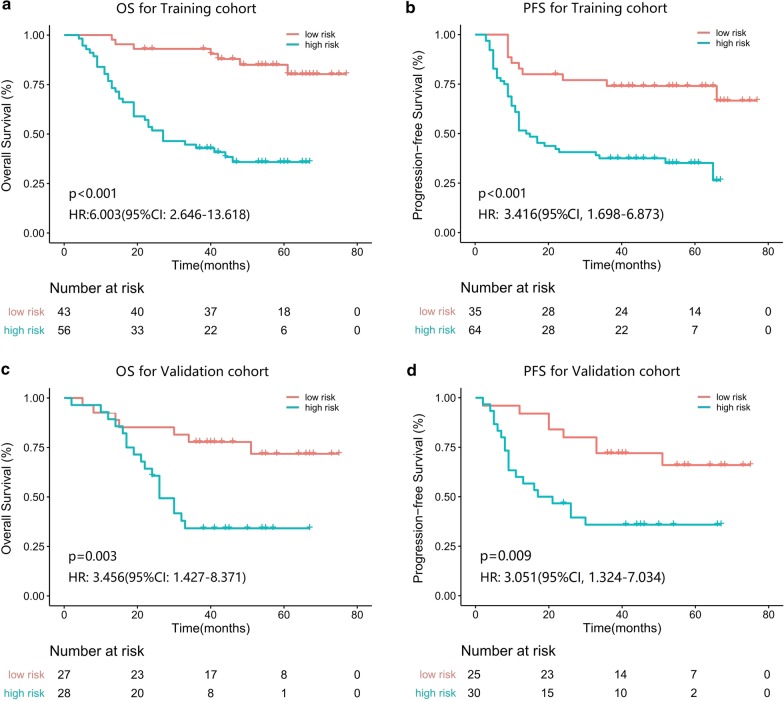
Kaplan–Meier survival analysis of overall survival and progression-free survival according to the optimum cut-offs of IBM scores. **a** Kaplan–Meier survival analysis of OS for the training cohort; **b** Kaplan–Meier survival analysis of PFS for the training cohort; **c** Kaplan–Meier survival analysis of OS for the validation cohort; **d** Kaplan–Meier survival analysis of PFS for validation cohort. We calculated the *p* value using the log-rank test. HR, hazard ratio; CI, confidence interval; IBM, imaging biomarker; OS, overall survival; PFS, progression-free survival

In the training cohort, we evaluated the predictive accuracy of the IBM score using ROCs analysis at different time points of follow-up. As shown in Fig. 3a, b, the area under the curves (AUCs) of the IBM score were 0.856 (95% CI 0.756–0.955, *p* < 0.001) and 0.779 (95% CI 0.663–0.895, *p* < 0.001) in terms of 5-year OS and 5-year PFS, respectively; and the optimal cut-off values of YI were 1.012 and 0.688, respectively. According to the maximum YI, the optimal cut-off values generated by ROC curves were 1.012 for 5-year OS and 0.688 for 5-year PFS. Patients were then stratified into high-risk or low-risk subgroups. In the training cohort, the 5-year OS and 5-year PFS were 85.0% and 74.1% respectively for the low-risk subgroup and 35.9% and 35.2% respectively for the high-risk subgroup (hazard ratios [HRs]:6.003 (95% CI 2.646–13.618) and 3.416 (95% CI 1.698–6.873), respectively; all *p* < 0.001, log-rank test; Fig. 4a, b). We then tested the same analyses using the ROC and Kaplan–Meier analysis, and similar results were observed in the validation cohort. As shown in Fig. 3c, d, the AUCs of the IBM score were 0.867 (95% CI 0.726–1.000, *p* = 0.001) and 0.852 (95% CI 0.713–0.990, *p* = 0.002) for the 5-year OS and 5-year PFS, respectively. Patients were then stratified into high-risk or low-risk subgroups. In the validation cohort the 5-year OS and 5-year PFS were 67.9% and 66.0% respectively for the low-risk subgroup; and 30.8% and 35.9% respectively for the high-risk subgroup (HR 2.957 (95% CI 1.104–7.919) and 3.051 (95% CI 1.324–7.034), respectively; all *p* < 0.05; Fig. 4c, d). Patients with OSCC with lower IBM scores were more likely to obtain a survival benefit from definitive CCRT. Those with high IBM scores had significantly poorer OS and PFS according to univariable Cox regression analysis (Additional file 1: Table S3). Multivariable Cox regression analysis for clinical factors and IBM score also revealed that the IBM score remained a powerful and independent predictive factor for OS and PFS in both training and validation cohorts (Table 2).

**Table 2:** Multivariable association of IBM score, clinical factors with OS and PFS in the training and validation cohort (likelihood Ratio: Backward stepwise)

Variables	Training cohort		Validation cohort	
	HR (95% CI)	*p*	HR (95% CI)	*p*
Overall survival				
IBM score	8.636 (3.572–20.876)	< 0.001	4.479 (1.707–11.750)	0.068
T stage*	0. 749 (0.423–1.325)	0.321	2.747 (1.403–5.376)	0.045
M stage*	1.072 (0.259–4.448)	0.923	1.522 (0.120–19.340)	0.746
Clinical stage*	1.345 (0.782–2.314)	0.284	0.679(0.283–1.632)	0.387
Progression-free survival				
IBM score	11.471 (3.123–42.134)	< 0.001	6.341 (1.667–24.115)	0.007
Age	0.991 (0.332–1.035)	0.681	0.962 (0.902–1.026)	0.238
Sex (female vs. male)	0.602 (0.283–1.280)	0.188	0.429 (0.123–1.499)	0.185
M stage*	1.917 (0.929–3.960)	0.078	0.824 (0.237–2.862)	0.761
Clinical stage*	1.006 (0.496–2.041)	0.986	1.484 (0.505–4.363)	0.473

IBM, imaging biomarker; CI, confidence interval; HR, hazard ratio

^*^According to American Joint Committee on Cancer (AJCC) staging system 6th

### Clinical benefit of IBM score

**Fig. 5:**
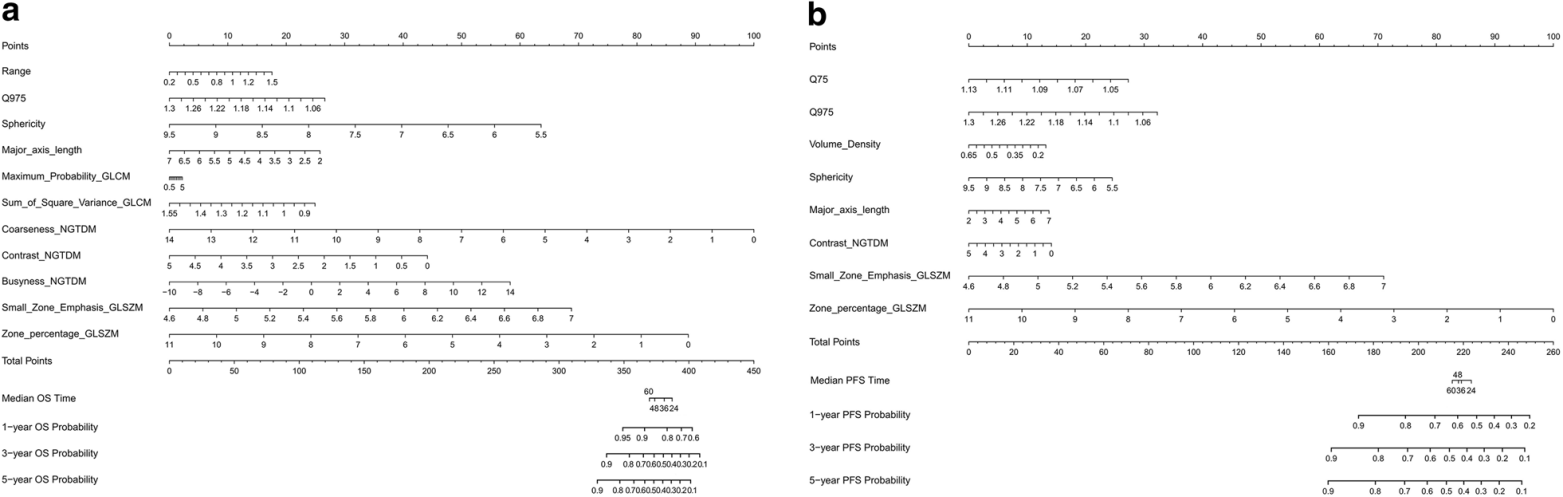
IBM nomograms for OS (**a**) and PFS (**b**). The constructed IBM nomograms were used to estimate OS and PFS for individual OSCC patients. IBM, imaging biomarker; OS, overall survival; PFS, progression-free survival; GLCM, grey level co-occurrence matrices; NGTDM, neighbourhood grey-tone difference matrices; GLSZM, grey level size-zone matrices

Clinical stage was associated with OS and PFS when using the Cox univariable analysis; however, it was not identified as a predictive independent factor for OS or PFS using multivariable analysis in the training cohort. IBM nomograms using only IBM scores for OS and PFS were constructed (Fig. 5a, b). In the training cohort, the C-indices of the models were 0.734 (95% CI 0.664–0.804) and 0.658 (95% CI 0.587–0.729) for OS and PFS, respectively. Similar results were observed in the validation cohort; the C-indices were 0.672 (95% CI 0.578–0.766) and 0.666 (95% CI 0.574–0.758) for OS and PFS, respectively. The C-index values showed that the IBM nomograms had good prognostic performance in both training and validation cohorts.

**Fig. 6:**
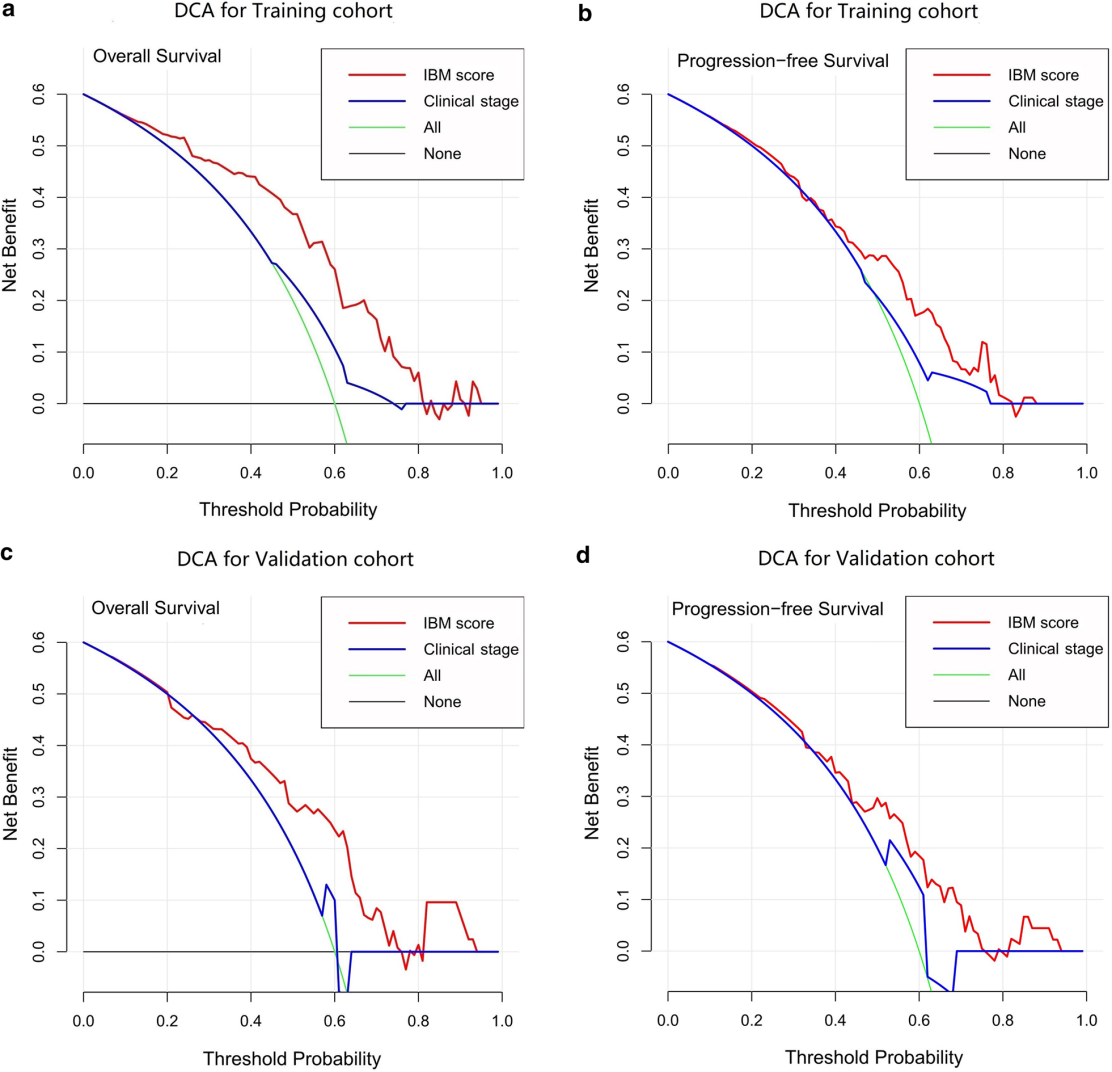
Decision curve analysis of PFS and OS were compared between the IBM score and clinical stage in the training and validation cohort, respectively. **a** DCA of OS for the training cohort; **b** DCA of PFS for the training cohort; **c** DCA of OS for the validation cohort; **d** DCA of PFS for the validation cohort. The y-axis represents the net benefit. The x-axis represents the threshold probability. Across the full range of threshold probabilities, the horizontal black line indicated that no patient chose to undergo follow-up, and the green line indicated that all patients underwent follow-up. The red line represented IBM score. The blue line represented clinical stage. Compared to the clinical stage, the IBM score had the higher net benefit. DCA, decision curve analysis; IBM, imaging biomarker; OS, overall survival; PFS, progression-free survival

The decision curve analysis showed that IBM score had higher overall net benefits than clinical stage, within a major range of reasonable threshold probability (Fig. 6). Compared to clinical stage, the IBM score demonstrated better discrimination capability in both training and validation cohorts.

## Discussion

This study showed that IBMs from contrast-enhanced CT images might allow prediction of OS and PFS for OSCC patients. The IBM score was revealed to be an independent prognostic factor for OSCC patients. Patients were successfully stratified into low-risk and high-risk subgroups by the IBM score, with significant differences in OS and PFS. IBM nomograms demonstrated better discrimination capability compared to traditional clinical stage, indicating the clinical value of the IBM score for individualised OS and PFS estimation.

The new IBM scores demonstrated significant associations with the OS and PFS of patients with OSCC. For geometric IBMs, sphericity, volume-density and the major axis length quantified the sphericity and size of tumours. Previous studies revealed that these IBMs basically represented tumour volume, which were significantly associated with treatment outcomes [30, 31]. In our study, the discrimination performances of the IBM nomograms were decreased when volume-related IBMs were omitted from the IBM score (C-index for the radiomics nomogram: OS, 0.672 (95% CI, 0.588–0.757); PFS, 0.629 (95% CI, 0.545–0.713) in the training cohort). These volume-related IBMs can promote the objective evaluation of subtle changes within tumours and provide clues to lesion invasiveness and growth-patterns [30, 32]. Range and Q975 were obtained from the histogram of voxel intensities and represented the heterogeneity of voxel intensities within the ROI [27].

The higher value texture IBMs, including maximum probability and sum of square variance, indicated the greater distribution variability of grey-level intensity values in the image [33, 34]. Coarseness, contrast, and busyness were all textural IBMs derived from NGTDM. Coarseness was used to quantify the granularity of the VOI of the tumour. Our study showed that higher value of busyness, lower values of coarseness or of contrast, might all be associated with poorer OS. The predictive and prognostic value of these pre-treatment IBMs had been previously demonstrated in several types of cancer [35–37]. Tixier et al. reported that coarseness from NGTDM was a strong predictor of treatment response for patients with OC following definitive CCRT [35]. However, IBMs derived from NGTDM were influenced by the reconstruction settings; therefore multicentre trials are still needed to standardise these IBMs [38]. Small zone emphasis measures the distribution of small size zones and small dependencies, while zone percentage assesses the distribution of large zones of the same intensity, and not of small groups of pixels or segments in any given direction [26, 35]. These texture IBMs containing spatial information among voxels could strongly reflect intra-tumour heterogeneity which was highly relevant to poor prognosis [12]. In order to correlate the multiple IBMs with the pathophysiological basis of tumours in an intuitive method, we constructed the multi-feature IBM score, which provided novel oncological biomarkers for obtaining phenotypic information, potentially assisting clinicians in formulating management strategies.

Current guidelines recommend definitive CCRT as a standard component for locally advanced OSCC therapies. However, several studies suggested that certain subgroups of patient failed to benefit from the present definitive CCRT strategies [6, 9]. Therefore, accurately distinguishing the risk subgroups of OSCC patients will help improve the current prognostic system and guide towards more personalised treatment. A few studies have focused on the correlations between radiomics analysis and treatment outcomes evaluation. Zhai et al. [30]. found that heterogeneous IBMs on CT images were significantly correlated with OS and helped improve the performance of clinical factors for OS among head and neck cancer patients. Mule et al. [39] investigated contrast-enhanced CT outcomes that might help predict survival in patients with advanced hepatocellular carcinoma treated with sorafenib. In the present study, our findings indicated that patients with OSCC with higher IBM scores had a greater likelihood of worse survival rates and failure to respond to CCRT. High-risk patients with OSCC identified in the present studies may benefit from more effective approaches to improve survival outcomes [40, 41]. Thus, the IBM score may serve as a prognostic tool for OSCC patients after definitive CCRT.

TNM staging is the most useful tool to stratify OSCC patients into different stages according to their tumour burden. However, its role in survival prediction among OSCC patients with the same clinical stage was non-significant. To develop an individualised easy-to-use tool for clinicians, we attempted to construct nomograms based on the IBM score to predict the prognosis of individual patients. These IBM nomograms could be used to predict the median survival time, and the probability of 1-year, 3-year and 5-year OS and PFS for individual OSCC patients. The nomograms performed well, with significant C-indices, and demonstrated good discrimination and clinical utility in both the training and validation cohorts. The decision curve analysis indicated that the IBM score was superior to the clinical stage, within a major range of reasonable threshold probability. Notably, time-dependent ROC curves showed that the IBM score did not have a good predictive performance for survival within 1 year. It was unclear why discrepancies remained for the training and validation cohorts. One possible explanation was the small sample size, retrospective nature of our study, and model-fitting differences. Further analysis for OSCC patients is needed to establish this.

For OC patients, contrast-enhanced CT scan is the main imaging procedure performed in conventional clinical practice [42]. It has been reported that IBMs extracted from contrast-enhanced CT images might be correlated with the spatial variability in microvessel density [43]. However, in standard CT images, IBMs might be associated with variability in tissue densities due to spatially variable fibrosis, cell density, and necrosis [13]. Badic at el. suggested that IBMs extracted from standard CT and contrast-enhanced CT images could provide complementary prognostic information from both approaches [44]. In view of the wide availability of contrast-enhanced CT scans among patients undergoing definitive radiotherapy, our study provides an important basis for conducting large-scale and multicentre research. It is important to note that quality assurance of contrast-enhanced CT scans will have a critical impact on radiomics based on these images. Furthermore, verification is needed on whether IBMs extracted from contrast-enhanced CT images could provide prognostic information for patients with oesophageal adenocarcinoma.

The limitations of our retrospective design include several aspects that were insufficient for the model [45]. This was a retrospective, single-centre study, involving a relatively small sample size. This could be addressed more thoroughly in future by using a larger sample size with multicentre validation cohorts to acquire high-level evidence for survival outcomes. Compared to the IBM score, the clinical factors used in this study demonstrated poor discrimination ability in predicting OS and PFS; other potential prognostic biomarkers could be incorporated into our IBM nomograms. A combination of multiple biomarkers and IBMs may improve the capability of predicting OS and PFS among patients with OSCC undergoing definitive CCRT.

## Conclusions

We demonstrated that IBMs extracted from contrast-enhanced CT images could effectively predict survival among OSCC patients. The IBM score might serve as a non-invasive predictive tool to guide individualised treatment decisions. Further studies with a larger sample size and multicentre validation are required.

## Supplementary information

**Additional file 1:** Supplementary Tables.

**Additional file 2:** Figure S1.

## Data Availability

All data supporting the conclusions of this article is available upon request from the corresponding author.

## Abbreviations

OSCC: Oesophageal squamous cell carcinoma;
CT: Computed tomography
IBMs: Imaging biomarkers
CCRT: Concurrent chemoradiotherapy
AJCC: American Joint Committee on Cancer (AJCC) staging system (version 6.0th)
SCC: Squamous cell carcinoma
PF: Cisplatin and 5-fluorouracil
IMRT: Intensity modulated radiation therapy
SMART: Simultaneous modulated accelerated radiotherapy
GLCM: Grey level co-occurrence matrices
GLRLM: Grey level run-length matrices
NGTDM: Neighbourhood grey-tone difference matrices
GLSZM: Grey level size-zone matrices
LASSO: Least absolute shrinkage and selection operator
VIF: Variance inflation factor
YI: Youden Index
DCA: Decision curve analysis
ROC: Receiver operation characteristic
AUC: Area under the curve
CI: Confidence interval
OS: Overall survival
PFS: Progression-free survival
