# Supplementary material for: Imaging biomarkers of contrast-enhanced computed tomography predict survival in oesophageal cancer after definitive concurrent chemoradiotherapy
Source: Radiat Oncol. 2021 Jan 12;16:8. doi: 10.1186/s13014-020-01699-w (PMC7805131; doi:10.1186/s13014-020-01699-w)
Supplement: Supplementary file 1 — Additional file 1: Supplementary Tables. [file 13014_2020_1699_MOESM1_ESM.docx]

**Supplementary Tables**

| Table S1. Univariable association of 18 IBMs with OS and 12 IBMs with PFS in the training cohort. | | | | | |
| --- | --- | --- | --- | --- | --- |
| Variables | Overall Survival | |  | Progression-free Survival | |
|  | HR (95%CI) | *p* |  | HR (95%CI) | *p* |
| Max | 1.003(1.000-1.007) | 0.089 |  | - | - |
| Range | 3.013(1.025-8.854) | 0.045 |  | - | - |
| Q025 | 0.999(0.997-1.000) | 0.135 |  | - | - |
| Q75 | - | - |  | 0.001(0.000-2.532) | 0.062 |
| Q975 | 0.001(0.000-1.984) | 0.062 |  | 0.001(0.000-0.143) | 0.019 |
| Volume_Density | 0.011(0.000-0.850) | 0.042 |  | 0.051(0.001-2.333) | 0.127 |
| Sphericity | 0.605(0.383-0.958) | 0.032 |  | 0.777(0.508-1.189) | 0.245 |
| Major_axis_length | 1.492(1.082-2.059) | 0.015 |  | 1.312(0.981-1.755) | 0.067 |
| Maximum_Probability_GLCM | 1.277(0.934-1.747) | 0.126 |  | 1.242(0.941-1.640) | 0.126 |
| Sum_of_Square_Variance_GLCM | 0.979(0.659-1.025) | 0.056 |  | - | - |
| Inverse_Difference_Normalized _san__GLCM | 2.239(0.736-6.812) | 0.156 |  | - | - |
| Coarseness_NGTDM | 0.749(0.510-1.101) | 0.141 |  | - | - |
| Contrast_NGTDM | 0.689(0.417-1.136) | 0.144 |  | 0.772(0.517-1.153) | 0.206 |
| Busyness_NGTDM | 1.136(0.993-1.300) | 0.063 |  | 1.084(0.955-1.229) | 0.212 |
| texture_strength_NGTDM | 0.289(0.069-1.222) | 0.092 |  | - | - |
| Small_Zone_Emphasis_GLSZM | 2.951(1.324-6.578) | 0.008 |  | 2.218(1.106-4.447) | 0.025 |
| Run_Length_Nonuniformity_ GLSZM | 1.001(0.999-1.003) | 0.243 |  | 1.001(0.999-1.003) | 0.193 |
| Zone_percentage_GLSZM | 0.748(0.515-1.088) | 0.129 |  | 0.771(0.561-1.058) | 0.107 |
| Small_Zone_Low_Grey_Level_Emphasis__GLSZM | 1.280(0.965-1.696) | 0.087 |  | 1.231(0.951-1.594) | 0.115 |

GLCM, grey level co-occurrence matrices; NGTDM, neighbourhood grey-tone difference matrices; GLSZM, grey level size-zone matrices

Table S2. The collinearity diagnosis for the 11 IBMs.

| Variables | Collinearity statistics | | | | |
| --- | --- | --- | --- | --- | --- |
|  | Overall Survival | |  | Progression-free Survival | |
|  | Tolerance | VIF |  | Tolerance | VIF |
| range | .455 | 2.196 |  | - | - |
| Q75 | - | - |  | 0.397 | 2.518 |
| Q975 | .294 | 3.403 |  | 0.221 | 4.524 |
| Volume_Density | - | - |  | 0.300 | 3.336 |
| Sphericity | .449 | 2.230 |  | 0.288 | 3.478 |
| Major_axis_length | .334 | 2.994 |  | 0.546 | 1.832 |
| Maximum_Probability_GLCM | .479 | 2.086 |  | - | - |
| Sum_of_Square_Variance_GLCM | .421 | 2.375 |  | - | - |
| Coarseness_NGTDM | .637 | 1.569 |  | - | - |
| Contrast_NGTDM | .670 | 1.492 |  | 0.475 | 2.107 |
| Busyness_NGTDM | .870 | 1.150 |  | - | - |
| Small_Zone_Emphasis_GLSZM | .656 | 1.525 |  | 0.790 | 1.266 |
| Zone_percentage_GLSZM | .374 | 2.672 |  | 0.474 | 2.110 |

VIF, variance inflation factor; GLCM, grey level co-occurrence matrices; NGTDM, neighbourhood grey-tone difference matrices; GLSZM, grey level size-zone matrices

| Table S3. Univatiate analysis for correlation of IBM score, clinical factors with OS and PFS in the training and validation cohort. | | | | | |
| --- | --- | --- | --- | --- | --- |
| Variables | Training cohort | |  | Validation cohort | |
|  | HR (95%CI) | *p* |  | HR (95%CI) | *p* |
| Overall Survival |  |  |  |  |  |
| **IBM score** | **8.636 (3.572-20.876)** | **<0.001** |  | **4.479 (1.707-11.750)** | **0.002** |
| Age | 0.980 (0.941-1.020) | 0.313 |  | 0.993 (0.937-1.052) | 0.804 |
| Sex (male vs. female) | 0.704 (0.312-1.585) | 0.396 |  | 0.203 (0.048-0.961) | 0.031 |
| Tumor location | 1.012 (0.639-1.602) | 0.959 |  | 0.523 (0.220-1.243) | 0.142 |
| T stage^*^ | 0.982 (0.527-1.827) | 0.234 |  | 2.747 (1.403-5.376) | 0.003 |
| N stage^*^ | 1.350 (0.710-2.566) | 0.361 |  | 0.874 (0.396-1.925) | 0.737 |
| M stage^*^ | 1.636 (0.726-3.685) | 0.235 |  | 0.458 (0.108-1.945) | 0.290 |
| **Clinical stage^*^** | **1.659 (1.033 -2.665)** | **0.036** |  | 1.095 (0.613-1.953) | 0.760 |
| Chemotherapy regimen | 1.074 (0.383-3.012) | 0.893 |  | 1.211 (0.362-4.048) | 0.756 |
| Dose regimen | 1.394 (0.760-2.557) | 0.283 |  | 1.538 (0.659-3.588) | 0.319 |
| Progression-free Survival | |  |  |  |  |
| **IBM score** | **11.471 (3.123-42.134)** | **<0.001** |  | **6.341 (1.667-24.115)** | **0.007** |
| Age | 0.973 (0.937-1.010) | 0.154 |  | 0.985 (0.932-1.042) | 0.606 |
| Sex (male vs. female) | 0.602 (0.283-1.280) | 0.188 |  | 0.302 (0.091-1.004) | 0.051 |
| Tumor location | 0.916 (0.606-1.387) | 0.680 |  | 0.673 (0.373-1.215) | 0.189 |
| T stage^*^ | 1.170 (0.769 -1.779) | 0.464 |  | 2.489 (1.344-4.612) | 0.004 |
| N stage^*^ | 1.248 (0.704-2.213) | 0.449 |  | 1.032 (0.478-2.224) | 0.937 |
| M stage^*^ | 1.939 (0.941-3.995) | 0.072 |  | 0.708 (0.213-2.352) | 0.573 |
| **Clinical stage^*^** | **1.616 (1.047-2.496)** | **0.030** |  | 1.278 (0.734-2.224) | 0.385 |
| Chemotherapy regimen | 0.943 (0.339-2.620) | 0.910 |  | 1.088 (0.327-3.614) | 0.891 |
| Dose regimen | 1.307 (0.758-2.254) | 0.335 |  | 1.494 (0.668-3.339) | 0.328 |

IBM, image biomarker; OS, overall survival; PFS, progression-free survival.

^*^ American Joint Committee on Cancer (AJCC) staging system (version 6.0th)
