# Supplementary figures and images for: Imaging biomarkers of contrast-enhanced computed tomography predict survival in oesophageal cancer after definitive concurrent chemoradiotherapy
Source: Radiat Oncol. 2021 Jan 12;16:8. doi: 10.1186/s13014-020-01699-w (PMC7805131; doi:10.1186/s13014-020-01699-w)

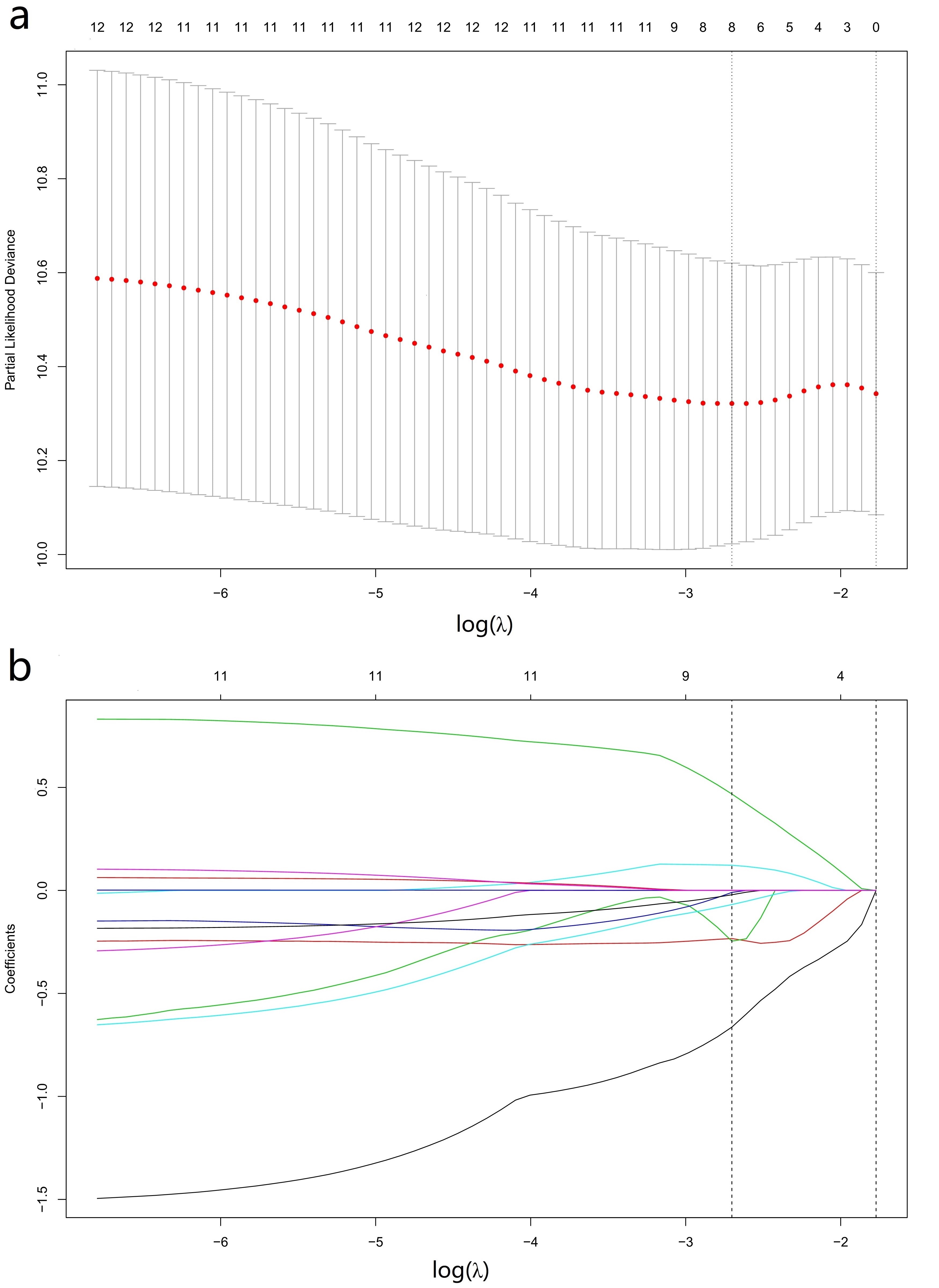

Supplement: Supplementary file 2 — Additional file 2: Figure S1. [file 13014_2020_1699_MOESM2_ESM.jpg]
